# Prevalence of non-contact and contact childhood sexual abuse: An Internet-based sample of men who have sex with men in China

**DOI:** 10.1371/journal.pone.0175444

**Published:** 2017-04-10

**Authors:** Wenjian Xu, Lijun Zheng, Yong Zheng

**Affiliations:** 1 Key Laboratory of Cognition and Personality (MOE), Southwest University, Chongqing, China; 2 Faculty of Psychology, Southwest University, Chongqing, China; Stellenbosch University, SOUTH AFRICA

## Abstract

**Background:**

The prevalence of childhood sexual abuse (CSA) is high among Western men who have sex with men (MSM), and CSA is associated with certain socio-demographic variables and with human immunodeficiency virus (HIV) status. Little is known about CSA among Chinese MSM; therefore, we explored the prevalence and frequency of non-contact (e.g., sexual invitations, exhibitionism) and contact (e.g., intercourse, genital contact) forms of CSA in an Internet-based sample of MSM in China. We also examined the associations between the participants’ socio-demographic characteristics and HIV status, and their histories of CSA.

**Methods:**

We surveyed MSM from 30 Chinese provinces in 2014–2015; 1,030 (mean age = 25.15 years, SD = 6.32) and 1,020 (mean age = 25.05 years, SD = 5.95) respondents were eligible for inclusion in the non-contact and contact CSA groups, respectively.

**Results:**

Prevalence of non-contact and contact CSA was 36.2% and 29.8%, respectively; 20.4% and 15.0% had experienced non-contact and contact CSA ≥ 3 times, respectively. Most respondents were young adults, well educated, single, had never “come out,” and self-identified as gay or bisexual men. Univariate analyses showed that respondents who had experienced contact CSA were more likely to be HIV-positive than those who had never experienced contact CSA. Multivariate analyses showed that non-contact CSA was associated with less education, being in a relationship with a woman or a man, and having “come out.” Contact CSA was associated with less education, higher income, and being in a relationship with a man. Respondents who had experienced non-contact CSA ≥ 3 times were more likely to be less educated, “out,” and to be in a relationship with a woman or a man. Respondents who had experienced contact CSA ≥ 3 times were more likely to be less educated and to be in a relationship with a man.

**Conclusions:**

It is necessary to pay closer attention to CSA among MSM and other sexual minorities in China.

## Introduction

People who experience childhood sexual abuse (CSA) are more likely to become infected with human immunodeficiency virus (HIV) [1–4]. Numerous studies have reported that sexual abuse has negative effects on individuals’ physical and mental health. Sexual minority groups, such as men who have sex with men (MSM), are more likely to report past experiences of CSA than heterosexual populations [5,6], and the prevalence of CSA among MSM ranges from 16% to 40% [2,3,7–9]. These discrepancies indicate ongoing inconsistencies in reports of the actual prevalence of CSA among MSM. The prevalence estimates of CSA among male sexual minorities have varied depending on the definitions of CSA that have been used (e.g., the specificity of the abusive behavior, the abused person’s age, and the perpetrator’s age). One study required the abused child to be younger than 12 years of age when the CSA occurred and the age difference between the abused child and perpetrator to be greater than 5 years [10]. Other studies have required only that the abused person be a child and the perpetrator be an adult [2,4,9]. The estimated prevalence of CSA among sexual minorities has been influenced by the year in which the data were collected and the sampling and recruitment strategies used in those studies [11–13]. Nevertheless, the prevalence of CSA among MSM is significantly higher than men in the general population, which implies that in Western nations, such as the United States, Canada, and Great Britain, and in Latin American countries, including Brazil, individuals at risk for becoming HIV-positive are more likely to have experienced CSA [2,6,14–16].

Increased rates of new HIV infections among MSM in China have attracted the attention of researchers and the Chinese government. The prevalence of HIV infection has not exceeded 1% of the general population and the proportion of those infected with HIV in China has been relatively low [17,18]. The spread of HIV among high-risk groups, including drug users and commercial blood donors, has progressed at a very slow rate [17–20]. However, among all reported cases of newly diagnosed HIV infections in 2014, the proportion of infections among MSM has increased from 0.7% in 2005, to 25.0% in 2014 [17,21]. The rapid rise in China’s prevalence of HIV infection among MSM [17,21,22] suggests an increase in high-risk sexual behaviors and a need to increase the use of safe sex practices. Based on self-reports of CSA, there is considerable evidence that CSA, is associated with high-risk sexual behaviors among MSM [2,4,8].

Research indicates that cultural beliefs, values, and norms might affect estimates of the prevalence of CSA [12]. Differences in the social acceptance of CSA and homosexuality might be underlying reasons for the variations in reporting practices, and thus, in prevalence rates [23]. For example, in Hispanic cultures, homosexual experiences among boys are considered taboo [24]. In collectivist societies, such as China, harmony with the social environment is highly valued. Individuals are typically oriented to the group, and their self-identities are deeply rooted in the groups with which they are attached, such as their families, classmates, and colleagues [25,26]. The groups’ needs might be more important than the needs of the individuals. Additionally, familial submissiveness and obedience are highly valued. If sexual abuse occurs within a family, non-disclosure protects the family from the shame of being investigated for perpetrating CSA. Many Chinese individuals follow the principle of stoicism, which places greater emphasis on developing pragmatic solutions than expressing feelings. The negative consequences of expressing one’s feelings might prevent a Chinese survivor of CSA from discussing the pain of the abuse and the distress of having survived it [24]. Therefore, CSA experiences are disclosed less frequently in collectivist cultures than they are in individualistic cultures [13].

The few research studies that have investigated CSA rates in China have mostly concentrated on CSA among women [27] and adolescents in the general population [28,29]. In a study by Chen and his colleagues, 21.9% of female college students in China reported that they had experienced CSA [28]. In another study that was conducted using a cross-sectional survey, 4.8% of Chinese female and male adolescents reported having sexual intercourse. Among them, 40.9% of the females and 29.6% of the males reported that they had been sexually coerced [29]. It should be noted that none of the studies conducted in China focused on MSM.

Given the high rate of CSA, its association with HIV status among MSM in Western cultures, and the rapid increase in HIV infection among MSM in China, related variables require examination in the Chinese social context. These variables include “coming out” status, a greater likelihood of being married [17], and China’s strategic plan to prioritize HIV-testing among MSM [30]. As stated earlier, MSM who report frequent CSA are more likely to be HIV-positive [2]. Moreover, actions that constitute CSA can affect the reporting behaviors of abused children, and thereby, the prevalence estimates of CSA. For example, non-contact forms of CSA, such as sexual invitations and exhibitionism have higher report rates and prevalence estimates than do contact forms of CSA, such as genital contact and intercourse. Differences between the definitions of these two types of CSA might account for the discrepancies in their prevalence estimates among MSM [21]. Furthermore, differences between their definitions in most Chinese [27,28,31,32] and some Western studies [33,34] should be examined. Therefore, we explored the prevalence and frequency of non-contact and contact CSA among an Internet-based sample of Chinese MSM. We also examined the association of CSA with socio-demographic characteristics and HIV status in this collectivist culture.

## Materials and methods

### Sample and recruitment

We conducted a cross-sectional study from 2014 to 2015 using a mixed-sampling approach, which included advertisements on websites (i.e., gay chat rooms from each province), software applications (i.e., Blued and Zank app software, which are popular among gay communities in China), and an instant messaging service (i.e., QQ, a popular service with chat groups in China). Data collection lasted approximately four months. Each day, from 8:00 a.m. to 12:00 p.m., we posted the same statement on all of the sites and an application for a personal account. Volunteers who expressed interest were invited to complete the questionnaire via a popular Chinese website that is widely used for professional surveys: Wenjuanxing (www.sojump.com). We excluded duplicate questionnaires based on our checks of the volunteers’ IP addresses.

In the beginning of the questionnaire, we provided a brief statement about the survey. Data were obtained from respondents who finished and submitted their online questionnaires. Personal information was not obtained if a respondent did not complete or submit the questionnaire. The initial dataset consisted of 1,191 respondents from 30 Chinese provinces that were eligible for enrollment. The inclusion criteria were as follows: (1) male, (2) aged 18 years or older, (3) living in China (the website showed the user’s city address), and (4) a history of having sex with another man at least once (sex was defined as oral or anal). We excluded respondents (n = 82) who did not meet the inclusion criteria. The final dataset consisted of 1,109 respondents, including 1,030 men in the non-contact CSA group (79 men with unknown non-contact CSA experiences were excluded) and 1,020 men in the contact CSA group (89 men with unknown contact CSA experiences were excluded).

### Ethics statement

Trained investigators explained the study’s aim to the volunteers before obtaining their Internet-based informed consent. At the beginning of the questionnaire, the respondents were informed that their participation in the survey was anonymous and voluntary, and that they had the right to withdraw from the study at any time. The Ethics Committee of Southwest University of China approved this study.

### Childhood sexual abuse (non-contact and contact)

Two questions pertaining to CSA were adopted from prior studies [4,27,35,36], including those conducted in China [27,32]. Three bilingual psychology professionals translated the questions into Chinese. Age differences were not accounted for in previous Chinese studies or those conducted outside of China [4,12,34]. Therefore, we included age in our definitions of both non-contact and contact CSA.

Non-contact CSA was measured by asking “Have you ever been forced or frightened by an adult(s) into doing something sexually that you did not want to do before you were 18 years old, including watching them expose their genitals to you, masturbating in front of you, or trying to sexually arouse you?”

Contact CSA was measured by asking “Have you ever been forced or frightened by an adult(s) into doing something sexually that you did not want to do before you were 18 years old, including touching someone or having them touch you sexually, engaging in mutual masturbation, or being forced or frightened into doing other types of sexual activities, including oral, anal, or vaginal sex?”

In response to these questions, participants selected one of five options (never, once, twice, three or more times, or refused to answer). Participants were classified as having had non-contact and contact CSA experiences if they answered once, twice, or three or more times. The participants’ responses were reclassified because the sample sizes of the non-contact and contact groups with the “once” and “twice” responses were too small to analyze the frequency of CSA and its associations [2]. The three new categories included “never had,” “once or twice,” and “three or more times.”

### Socio-demographic characteristics

The participants’ socio-demographic characteristics included their region of China, age, sex, highest educational level (less than high school, high school, college, or postgraduate or more), employment status (student, employed, or unemployed), sexual orientation (gay, bisexual, heterosexual, or other), and current relationship status (relationship with a woman, relationship with a man, or single).

Participants’ monthly salary (< ¥2,000/$323, ¥2,000/$323–3,999/$645, ¥4,000/$645–5,999/$968, ¥6,000/$968–9,999/$1,613, or ≥ ¥10,000/$1,613) was measured using China’s income-level ranges [37].

Participants were asked whether they had ever “come out” (never, partially, or fully). This question was adapted from previous studies [38,39] to measure the extent of the respondents’ disclosures of their sexual orientation to others.

Participants were also asked whether they had ever been tested for HIV; if they answered yes, they were asked about the number of times they were tested for HIV within the last 6 months (never, once or twice, or three or more times). Increasing HIV detection among MSM is a priority strategic plan in China. There is a growing number of studies on this topic [30]; therefore, we examined whether CSA was associated with HIV testing.

### HIV status

Participants were asked whether they had been infected with HIV (negative, positive, or unsure).

### Statistics

Data were analyzed using SPSS 17.0 for Windows. We calculated the prevalence rates and frequencies of non-contact and contact CSA. Participants who refused to answer the questions about their CSA experiences were excluded from the data analysis. The respondents reported their frequencies of non-contact (N = 1,030) and contact CSA (N = 1,020). We divided the respondents into two groups based on whether they had experienced CSA. We also tabulated socio-demographic characteristics and HIV status by participants’ reported frequency of CSA: (1) once or twice and (2) three or more times. A chi-square test was used to compare the associations between the socio-demographic characteristics and HIV status with CSA experience. An independent-samples *t-*test and one-way analysis of variance were conducted to compare the association of age (continuous variable) and CSA. The level of statistical significance was set at *p* < .05. If the overall test was significant, a Bonferroni correction was performed to compare each level of the demographic variables. Furthermore, all variables were included in separate multivariate logistic regression models to determine the correlates of CSA. All logistic regression odds ratios (ORs) were reported using 95% confidence intervals (CIs).

## Results

### Socio-demographics

The mean age of participants with non-contact forms of CSA was 25.15 years old (SD = 6.32). The mean age of participants with contact forms of CSA was 25.05 years old (SD = 5.95). The numbers of cases of non-contact and contact CSA in the five regions of China are presented in Table 1, which also summarizes the socio-demographic characteristics of the participants with both forms of CSA. In the non-contact CSA group, most of the participants were single (62.6%), well educated (73.6% had a college degree at least), young adults (85.8% were younger than 30 years of age), not students (69.9% were either employed or unemployed), self-identified as gay/bisexual men (87.6%), and had never “come out” (64.5%). The socio-demographic characteristics of the contact CSA group were similar to those in the non-contact CSA group. Most of them were single (63.0%), well educated (74.2% had a college degree at least), young adults (86.1% were younger than 30 years of age), not students (69.9% were either employed or unemployed), self-identified as gay/bisexual men (87.7%), and had never “come out” (64.8%).

**Table 1:** Socio-demographic characteristics and HIV status of the study sample.

	N-CSA: N (%)			C-CSA: N (%)		
	No	Yes	Statistic	No	Yes	Statistic
	657 (63.8)	373 (36.2)		716 (70.2)	304 (29.8)	
**Characteristics**						
Region			1.68			0.93
East China	134 (20.4)	66 (17.7)	-	138 (19.3)	58 (19.1)	-
South China	82 (12.5)	48 (12.9)	-	94 (13.1)	35 (13.5)	-
West China	243 (37.0)	138 (37.0)	-	267 (37.3)	113 (37.2)	-
North China	45 (6.8)	31 (8.3)	-	50 (7.0)	25 (8.2)	-
Central China	153 (23.3)	90 (24.1)	-	167 (23.3)	73 (24.0)	-
Age M (SD)	24.9 (6.2)	25.6 (6.5)	-1.70	24.7 (5.6)	25.8 (6.6)	-2.62**
18–20 years	136 (20.7)	76 (20.4)		156 (21.8)	53 (17.4)	
21–25 years	309 (47.0)	152 (40.8)		333 (46.5)	129 (42.4)	
26–30 years	138 (21.0)	73 (19.6)		145 (20.3)	62 (20.4)	
> 30 years	74 (11.3)	72 (19.3)		82 (11.5)	60 (19.7)	
Educational level			10.95***			7.33**
High school or less	151 (23.0)	121 (32.4)		168 (23.5)	96 (31.6)	
College or more	506 (77.0)	252 (67.6)		548 (76.5)	208 (68.4)	
Occupation			0.10			4.06
Student	200 (30.4)	110 (29.5)		229 (32.0)	78 (25.7)	
Not student	457 (69.6)	263 (70.5)		487 (68.0)	226 (74.3)	
Monthly income			3.21			11.17**
< ¥2,000	234 (35.6)	123 (33.0)	-	267 (37.3)	91 (29.9)	10.01**
¥2,000–3,999	221 (33.6)	115 (30.8)	-	237 (33.1)	91 (29.9)	5.85*
≥ ¥4,000^a^	202 (30.7)	135 (36.2)	-	212 (29.6)	122 (40.1)	
Coming out status			7.68**			4.03*
Never	444 (67.6)	220 (59.0)		478 (66.8)	183 (60.2)	
Partially/fully	213 (32.4)	153 (41.0)		238 (33.2)	121 (39.8)	
Relationship status			19.32***			13.23***
With a woman	74 (11.3)	64 (17.2)	11.75***	86 (12.0)	49 (16.1)	6.13*
With a man	139 (21.2)	108 (29.0)	12.45***	153 (21.4)	89 (29.3)	10.30***
Single^a^	444 (67.6)	201 (53.9)		477 (66.6)	166 (54.6)	
Sexual orientation			1.18			0.55
Gay	432 (65.8)	249 (66.8)	-	474 (66.2)	205 (67.4)	-
Bisexual	138 (21.0)	83 (22.3)	-	150 (20.9)	65 (21.4)	-
Heterosexual/other	87 (13.2)	41 (11.0)	-	92 (12.8)	34 (11.2)	-
HIV testing			1.00			2.99
Never had	436 (66.4)	236 (63.3)		483 (67.5)	188 (61.8)	
Yes	221 (33.6)	137 (36.7)		233 (32.5)	116 (38.2)	
**HIV status**			3.99			6.03*
Negative	564 (85.8)	307 (82.3)	-	610 (85.2)	252 (82.9)	5.96*
Unsure	78 (11.9)	50 (13.4)	-	91 (12.7)	37 (12.2)	4.90
Positive^a^	15 (2.3)	16 (4.3)	-	15 (2.1)	15 (4.9)	

Notes: Data are presented as frequencies and (percentages). N-CSA = non-contact childhood sexual abuse; C-CSA = contact childhood sexual abuse.

* *p* < .05

** *p* < .01

*** *p* < .001.

^a^ The reference group in the post hoc test.

### HIV status

In the non-contact CSA group, 3.0% were HIV-positive and 12.4% were unsure of their HIV status. In the contact CSA group, 2.9% were HIV-positive and 12.5% were uncertain of their HIV status (Table 1).

### Frequency of childhood sexual abuse

Table 2 presents the frequency of non-contact CSA among the participants. In this sample, 373 (36.2%) reported having a history of non-contact CSA. Among them, 163 had experienced non-contact CSA once or twice and 210 had experienced non-contact CSA three or more times. Table 3 presents the frequency of contact CSA. In this sample, 304 (29.8%) reported having a history of contact CSA. Among them, 151 had experienced contact CSA once or twice and 153 had experienced contact CSA three or more times.

**Table 2:** Frequency of non-contact childhood sexual abuse among the participants by socio-demographic characteristics and HIV status (N = 1,030).

	Frequency of non-contact childhood sexual abuse			Statistic
	None	Once/Twice	≥ Three times	
	657 (63.8)	163 (15.8)	210 (20.4)	
**Characteristics**				
Region				10.16
East China	134 (20.4)	28 (17.2)	38 (18.1)	
South China	82 (12.5)	22 (13.5)	26 (12.4)	
West China	243 (37.0)	71 (43.6)	67 (31.9)	
North China	45 (6.8)	13 (8.0)	18 (8.6)	
Central China	153 (23.3)	29 (17.8)	61 (29.0)	
Age M (SD) in years	24.9 (6.2)	24.6 (5.6)	26.3 (7.1)	4.85**
18–20	136 (20.7)	40 (24.5)	36 (17.1)	
21–25	309 (47.0)	71 (43.6)	81 (38.6)	
26–30	138 (21.0)	25 (15.3)	48 (22.9)	
> 30	74 (11.3)	27 (16.6)	45 (21.4)	
Educational level				11.50**
High school or less	151 (23.0)	56 (34.4)	65 (31.0)	
College or more	506 (77.0)	107 (65.6)	145 (69.0)	
Occupation				3.36
Student	200 (30.4)	56 (34.4)	54 (25.7)	
Not student	457 (69.6)	107 (65.6)	156 (74.3)	
Monthly income				6.46
< ¥2,000	234 (35.6)	59 (36.2)	64 (60.5)	
¥2,000–3,999	221 (33.6)	53 (32.5)	62 (29.5)	
≥ ¥4,000^a^	202 (30.7)	51 (31.3)	84 (40.0)	
Coming out status				9.31**
Never	444 (67.6)	102 (62.6)	118 (56.2)	
Partially/fully	213 (32.4)	61 (37.4)	92 (43.8)	
Relationship status				26.94***
With a woman	74 (11.3)	26 (16.0)	38 (18.1)	
With a man	139 (21.2)	37 (22.7)	71 (33.8)	
Single^a^	444 (67.6)	100 (61.3)	101 (48.1)	
Sexual orientation				2.12
Gay	432 (65.8)	105(64.4)	144 (68.6)	
Bisexual	138 (21.0)	40 (24.5)	43 (20.5)	
Heterosexual/other	87 (13.2)	18 (11.0)	23 (11.0)	
HIV testing				1.00
Never had	436 (66.4)	103 (63.2)	133 (63.3)	
Yes	221 (33.6)	60 (36.8)	67 (36.7)	
**HIV status**				5.91
Negative	564 (85.8)	138 (84.7)	169 (80.5)	
Unsure	78 (11.9)	20 (12.3)	30 (14.3)	
Positive	15 (2.3)	5 (3.1)	11 (5.2)	

* *p* < .05

** *p* < .01

*** *p* < .001.

^a^ The comparison group in the post hoc test.

**Table 3:** Frequency of contact childhood sexual abuse among the participants by socio-demographic characteristics and HIV status (N = 1,020).

	Frequency of contact childhood sexual abuse			Statistic
	None	Once/Twice	≥ Three times	
	716 (70.2)	151 (14.8)	153 (15.0)	
**Characteristics**				
Region				5.93
East China	138 (19.3)	34 (22.5)	24 (15.7)	
South China	94 (13.1)	17 (11.3)	18 (11.8)	
West China	267 (37.3)	58 (38.4)	55 (35.9)	
North China	50 (7.0)	13 (8.6)	12 (7.8)	
Central China	167 (23.3)	29 (19.2)	44 (28.8)	
Age M (SD) in years	24.7 (5.6)	25.7 (6.3)	25.9 (6.9)	3.52*
18–20	156 (21.8)	24 (15.9)	29 (19.0)	
21–25	333 (46.5)	71 (47.0)	58 (37.9)	
26–30	145 (20.3)	28 (18.5)	34 (22.2)	
> 30	82 (11.5)	28 (18.5)	32 (20.9	
Educational level				7.33*
High school or less	168 (23.5)	48 (31.8)	48 (31.4)	
College or more	548 (76.5)	103 (68.2)	105 (68.6)	
Occupation				4.16
Student	229 (32.0)	40 (26.5)	38 (24.8)	
Not student	487 (68.0)	111 (73.5)	115 (75.2)	
Monthly income				11.95*
< ¥2,000	267 (37.3)	47 (31.1)	44 (28.8)	
¥2,000–3,999	237 (33.1)	47 (31.1)	44 (28.8)	
≥ ¥4,000^a^	212 (29.6)	57 (37.7)	65 (42.5)	
Coming out status				4.03
Never	478 (66.8)	91 (60.3)	92 (60.1)	
Partially/fully	238 (33.2)	60 (39.7)	61 (39.9)	
Relationship status				15.27**
With a woman	86 (12.0)	26 (17.2)	23 (15.0)	
With a man	153 (21.4)	39 (25.8)	50 (32.7)	
Single^a^	477 (66.6)	86 (57.0)	80 (52.3)	
Sexual orientation				2.40
Gay	474 (66.2)	97(64.2)	108 (70.6)	
Bisexual	150 (20.9)	37 (24.5)	28 (18.3)	
Heterosexual/other	92 (12.8)	17 (11.3)	17 (11.1)	
HIV testing				3.66
Never had	483 (67.5)	90 (59.6)	98 (64.1)	
Yes	233 (23.5)	61 (40.4)	55 (35.9)	
**HIV status**				6.15
Negative	564 (85.8)	126 (83.4)	126 (82.4)	
Unsure	78 (11.9)	18 (11.9)	19 (12.4)	
Positive	15 (2.3)	7 (4.6)	8 (5.2)	

* *p* < .05

** *p* < .01.

^a^ The comparison group in the post hoc test.

### Risk factors of childhood sexual abuse

Compared with respondents who had never experienced non-contact CSA, those who had experienced non-contact CSA were significantly less likely to be well educated (a college degree at least vs. a high school degree at most, χ^2^ = 10.95; *p* < .001) and significantly less likely to conceal their sexual orientation (χ^2^ = 7.68; *p* < .01). In addition, participants who had experienced non-contact CSA were significantly more likely to be in a relationship with a woman (vs. being single, χ^2^ = 11.75; *p* < .001; Bonferroni correction *p* = .017) or a man (vs. being single, χ^2^ = 12.45; *p* < .001; Bonferroni correction *p* = .017), compared with those who had never experienced non-contact CSA. In contrast, no association was found between non-contact CSA and HIV status, region, employment status, monthly income, sexual orientation, or the frequency of HIV testing. Compared with respondents who had never experienced contact CSA, those who had experienced contact CSA were significantly more likely to be HIV-positive (vs. HIV-negative, χ^2^ = 5.96; *p* = .015; Bonferroni correction *p* = .017). Participants who had experienced contact CSA were significantly less likely to be well educated (a college degree at least vs. a high school degree at most, χ^2^ = 7.33; *p* < .01) and significantly less likely to conceal their sexual orientation (χ^2^ = 4.03; *p* < .05), compared with those who had never experienced contact CSA. In addition, those with contact CSA experience were significantly more likely to be older (*t* = -2.62; *p* < .01), significantly more likely to have a higher monthly income (≥ ¥4,000 vs. < ¥2,000, χ^2^ = 10.01; *p* = .002; and ≥ ¥4,000 vs. ¥2,000–3,999, χ^2^ = 5.85; *p* = .016; Bonferroni correction *p* = .017), and significantly more likely to be in a relationship with a woman (vs. being single, χ^2^ = 6.13; *p* = .013; Bonferroni correction *p* = .017) or a man (vs. being single, χ^2^ = 10.30; *p* < .001; Bonferroni correction *p* = .017), compared with those who had never experienced contact CSA. In contrast, contact CSA was not associated with the participants’ region, employment status, sexual orientation, or frequency of HIV testing.

Table 4 presents the results of the multivariate analysis of the participants’ demographic characteristics associated with CSA. The participants’ experience with non-contact CSA was significantly and positively associated with a low level of education (vs. a college degree at least, Wald = 9.23, AOR = 1.56, *p* < .01), partial or full disclosure of their sexual orientation (Wald = 6.59, AOR = 1.42, *p* < .01), and currently being in a relationship with a woman (vs. being single, Wald = 10.99, AOR = 1.90, *p* < .001) or a man (vs. being single, Wald = 10.61, AOR = 1.66, *p* < .001). Their experience with contact CSA was significantly and positively associated with a low level of education (vs. a college degree at least, Wald = 7.21, AOR = 1.52, *p* < .01), a higher monthly income (< ¥2,000 vs. ≥ ¥4,000, Wald = 5.18, AOR = 0.67, *p* < .05; ¥2,000–3,999 vs. ≥ ¥4,000, Wald = 5.80, AOR = 0.66, *p* < .05), and currently being in a relationship with a man (vs. being single, Wald = 8.17, AOR = 1.60, *p* < .01).

**Table 4:** Multivariate analysis of the socio-demographic characteristics associated with N-CSA and C-CSA in a Chinese sample of MSM.

Model	N-CSA (N = 1,030)		C-CSA (N = 1,020)	
	Wald	AOR (95% CI)	Wald	AOR (95% CI)
Low educational level^a^	9.23	1.56 (1.17,2.08)**	7.21	1.52 (1.12, 2.07)**
Monthly income^b^				
< ¥2,000			5.18	0.67 (0.48, 0.95)*
¥2,000–3,999			5.80	0.66 (0.47, 0.93)*
"Out" as MSM^c^	6.59	1.42 (1.09,1.86)**		
Relationship status^d^				
With a woman	10.99	1.90 (1.30,2.77)***	2.35	1.38 (0.91, 2.09)
With a man	10.61	1.66 (1.22,2.26)***	8.17	1.60 (1.16, 2.20)**

Notes: N-CSA = non-contact childhood sexual abuse; C-CSA = contact childhood sexual abuse; AOR = adjusted odds ratio; CI = confidence interval.

* *p* < .05

** *p* < .01

*** *p* < .001.

^a^ College or higher is the reference group.

^b^ Monthly income greater than ¥4,000 is the reference group.

^c^ Never "out" as MSM is the reference group

^d^ Single is the reference group.

### Risk factors of frequency of childhood sexual abuse

Table 2 presents the associations between the frequency of non-contact CSA and the sample’s socio-demographic characteristics. Compared with men who reported they had not experienced non-contact CSA, those who had experienced non-contact CSA three or more times were significantly older (*F* = 4.85; *p* < .01; post hoc: *p* < .01), significantly more likely to have a low level of education (vs. a college degree at least, χ^2^ = 11.50; *p* < .01), significantly more likely to disclose their sexual orientation partially or fully (χ^2^ = 9.31; *p* < .01), and significantly more likely to be in a relationship with a woman (vs. being single, χ^2^ = 13.20; *p* < .001; Bonferroni correction *p* = .006) or a man (vs. being single, χ^2^ = 20.11; *p* < .001; Bonferroni correction *p* = .006). Compared with men who had not experienced non-contact CSA, those who had experienced non-contact CSA once or twice were significantly more likely to have a low level of education (vs. a college degree at least, χ^2^ = 8.95; *p* = .003; Bonferroni correction *p* = .017).

Table 3 presents the associations between the frequency of contact CSA and participants’ socio-demographic characteristics. Compared with men who had not experienced contact CSA, those who had experienced contact CSA three or more times were significantly more likely to be older (*F* = 3.52; *p* < .05; post hoc: *p* < .05), significantly more likely to have a higher monthly income (≥ ¥4,000 vs. < ¥2,000, χ^2^ = 8.42; *p* = .004; Bonferroni correction *p* = .006), and significantly more likely to be in a relationship with a man (vs. being single, χ^2^ = 11.06; *p* < .001; Bonferroni correction *p* = .006). Compared with men who reported they had not experienced contact CSA, those who had experienced contact CSA once or twice were significantly more likely to have a low level of education (vs. a college degree at least, χ^2^ = 5.40; *p* = .02; Bonferroni correction *p* = .017).

We also conducted a multivariate analysis of the variables that were associated with the frequency of participants’ experiences of non-contact and contact CSA, respectively. Having experienced non-contact CSA three or more times was significantly and positively associated with a low level of education (vs. a college degree at least, Wald = 4.21, AOR = 1.45, *p* < .05), partial or full disclosure of their sexual orientation (Wald = 7.47, AOR = 1.58, *p* < .01), and being in a relationship with a woman (vs. being single, Wald = 12.82, AOR = 2.29, *p* < .001) or a man (vs. being single, Wald = 17.10, AOR = 2.15, *p* < .001). Having experienced non-contact CSA once or twice was significantly and positively associated with a low level of education (vs. a college degree at least, Wald = 7.98, AOR = 1.71, *p* < .01). Furthermore, having experienced contact CSA three or more times was significantly and positively associated with a low level of education (vs. a college degree at least, Wald = 3.97, AOR = 1.48, *p* < .05) and being in a relationship with a man (vs. being single, Wald = 10.94, AOR = 1.96, *p* < .01). Having experienced contact CSA once or twice was significantly and positively associated with a low level of education (vs. a college degree at least, Wald = 4.02, AOR = 1.49, *p* < .05).

## Discussion

This is the first study to explore the prevalence of CSA and examine the associations between CSA and HIV status in an Internet-based sample of MSM in China. Among the respondents, 36.2% reported a history of non-contact CSA and 29.8% reported a history of contact CSA. Approximately half of the respondents who reported a history of CSA had experienced CSA three or more times. In this study, data were collected using an Internet-based sampling approach across the entire geographic region of China to obtain a sample from the MSM population, which has an alarmingly high prevalence of CSA.

This study’s results support the claim that CSA is a global problem of considerable magnitude. Consistent with studies conducted in Western nations and Latin America [2,3,6–9,14–16], the prevalence of CSA among MSM in this study indicates that CSA among male sexual minorities in China cannot be ignored. However, the prevalence estimates of CSA have been reported to vary according to the CSA definitions that are used, which may differ with respect to their specificity in Western countries [11,13]. Previous studies have also reported that low CSA rates in Asia might be found in studies because CSA is disclosed less often, regardless of gender, which might reflect the region’s collectivist rather than individualistic culture [13,27,28]. When the perpetrator and the abused child are from the same family, the collectivist cultural tradition of maintaining family unity and peace might negatively affect the child’s willingness to disclose the abuse. When the perpetrator is outside the abused child’s family, the child’s awareness of the consequences of reporting the abuse might prevent his/her disclosure of CSA [24].

Nevertheless, this study found high prevalence rates of CSA among Chinese MSM, which was unexpected. A possible reason for this finding is that sample-based Internet surveys are self-selective (i.e., some respondents who experienced CSA and were unwilling to disclose the CSA experience, withdrew from the study), and this method might ease the burden of disclosure for Chinese respondents. Additionally, recall bias might exist. Future studies are necessary to examine these explanations. A higher proportion of CSA was reported by study participants at sexually transmitted disease clinics [7,9]; therefore, we suggest that additional samples of MSM in China should be studied to gain a better understanding of CSA in this population.

Our findings are partially consistent with separate studies that have reported associations between contact CSA and HIV status [2,6,14]. We used an Internet-based sample of Chinese MSM, which indicated there was no association between CSA and HIV status in this collectivist culture, and suggested a possible cross-cultural commonality. We also found that the lower prevalence of HIV infection found in this study might have led to low statistical power to find an association between HIV status and CSA. Further studies using different data collection methods, such as face-to-face interviews and clinical records, are needed to examine this association. Given the study’s findings of an association between contact CSA and HIV status, it is necessary to take into account the individual’s history of contact CSA when implementing HIV interventions for MSM in China.

The regions represented in this sample included almost all the Chinese provinces. Most of the respondents identified themselves as gay or bisexual, well educated (a college degree at least), and single. The median age of the sample was similar to that of other Internet-based investigations in China [40]. Approximately 3% of the respondents were HIV-positive and 12.4% were unsure of their HIV status.

The demographic characteristics of the study groups were associated with the respondents’ history and frequency of experiencing CSA. Similar to prior studies [41,42], the probability of both non-contact and contact CSA was expected to decline with an increase in educational level, which was found to be a key variable associated with CSA. Another perspective is that having a history of CSA might hamper academic achievement. Possible interventions, such as education, counseling, and HIV testing should target those with low levels of education.

Interestingly, the respondents’ CSA experiences appeared to have been a reason for their “coming out.” Chinese gay/bisexual men have been reported to be unwilling to disclose their same-sex encounters because of societal stigma [17,43]; therefore, “coming out” status was associated with non-contact CSA in the present study.

In addition, we did not expect to find that respondents with a higher monthly income would be more likely to report contact CSA, but we did. This finding is inconsistent with that of a prior study [41], in which men who reported CSA were found to be more likely to have a lower income. We thought income might be confounded with age or employment status; for example, those earning less than ¥2,000 were also more likely to be students and younger. Compared with MSM who reported having no experience with non-contact CSA, those who reported experiencing non-contact CSA three or more times, were more likely to have less than a college degree and to disclose their sexual orientation. Compared with MSM who reported experiencing non-contact CSA, those who reported experiencing non-contact CSA three or more times were significantly more likely to be less educated (a high school degree at most).

Being single was associated with fewer occurrences of non-contact CSA, compared with being in a relationship with a man or a woman. Current attitudes about homosexuality and MSM in China are unfriendly, and most of the social pressure originates with friends and family because they fear “losing face” or having their reputations damaged [17]. One method of coping with these negative attitudes is by forming a relationship with a woman [44]. The participants who had a current relationship with a woman or a man during this study confirmed that they were more likely to be involved in a non-contact CSA experience. In addition, participants who reported contact CSA tended to be HIV-positive. Therefore, interventions, such as targeting online chat rooms and websites frequently accessed by MSM, are needed to promote condom use and general sex education in China.

Several limitations of this study should be noted. First, the data presented are cross-sectional; therefore, causal relationships cannot be determined. Second, the Internet-based sampling method might have led to sampling bias. The Internet respondents were self-selected; therefore, the collected data were representative of the individuals who were willing to answer questions requiring disclosure of personal information. However, given the difficulties associated with collecting data in China using face-to-face interviews, the Internet-based method is a useful alternative. Third, this study shares a shortcoming common among studies with similar participant groups (i.e., a primarily young MSM sample is more likely to be recruited than an older sample). Fourth, HIV infection is stigmatized and some MSM might be less likely to report their HIV-positive status; for example, a previous study [20] that used respondent-driven interviews reported an HIV-positive prevalence estimate of 6.1% among MSM in 2011. Fifth, monthly salary is determined by employment status, educational level, and age; therefore, bias could exist.

This study provides important data on the prevalence and the frequency of non-contact and contact CSA and their associations with HIV status and the socio-demographic characteristics of an Internet-based sample of MSM in China. These findings are based on socio-demographic variables in the cultural context of collectivism and can inform the source population from which the MSM sample was drawn. Finally, these findings confirm the importance of considering the prevalence of CSA and its effects on the delivery of prevention interventions and healthcare services to MSM in China.

## Supporting information

S1 Supplementary Material Dataset of Chinese sample of men who have sex with men for this study. (SAV)
